# American Dental Society of Europe

**Published:** 1887-12

**Authors:** E. A. Galbreath

**Affiliations:** Hannover


### AMERICAN DENTAL SOCIETY OF EUROPE.

### FIFTEENTH ANNUAL MEETING AT COBLENTZ, GERMANY, SEPTEMBER, 1887.

REPORTED FOR THE INDEPENDENT PRACTITIONER BY DR. E. A. GALBREATH, HANNOVER.

The fifteenth annual meeting of the American Dental Society of Europe was held in Coblentz, at the Hotel zum Riesen, commencing on the 1st day of September, 1887. The meeting was called to order by the President, Dr. George, at 11.30 o'clock.

The Secretary's report of the last meeting, held in Frankfort, was read and adopted.

The Committee on Membership proposed the following names to be voted upon at the next meeting :

Dr. Charles Henry Abbot, Berlin; Dr. Fred Merril, Milan; Dr. J. F. Patterson, Montreux.

The following gentlemen were then elected members of the Society:

Dr. Chas. H. Adams, Frankfort on the Main; Dr. Elof Förberg, Stockholm; Dr. George Hofman, Wiesbaden; Dr. H. C. Merril, Cologne.

Dr. Elliot announcing that on account of the two years' absence law he was no longer a member of the Society, it was resolved that his excuses be accepted, and his name again placed upon the roll of membership.

A committee consisting of Drs. Bryan, of Basel, and Jenkins, of Dresden, was appointed to arrange for a dinner at the hotel.

Adjourned to meet at 2 P. M.

### AFTERNOON SESSION.

Dr. Cunningham's paper on "Dental Education" was read by Dr. Elliot, Dr. Cunningham being absent, in attendance at the International Congress.

### DISCUSSION.

Dr. Jenkins called on Dr. Miller to give the relations of the Zahnarzt and the Zahnteckniker.

*Dr. Miller*—The authorized physician in Germany is required to have graduated from the Gymnasium. The authorized dentist must have become a Primaner, or have studied two years less. To be a Zahnteckniker, no preparation whatever is required by law. The dental student (Zahnarzt) must attend two Semesters in the dental department of a University, and then present himself for examination. This examination comprises:

FIRST—The diagnosing of practical cases in the mouth, lasting thirty minutes, upon which cases he writes out his treatment of the same. Time, three to six hours.

SECOND—The written examination, lasting six, ten or twelve hours.

THIRD—The practical examination, such as making fillings, sets of teeth on rubber and gold, and extractions.

If the restrictions imposed upon the Zahnarzt were greater, he could not compete with the Zahnteckniker, who on account of the German law which allows freedom of occupation (Gewerbefreiheit), practices with no restriction whatever. Our chief object in the University of Berlin has been to elevate the standard of practical work. Most of the dental students in Germany are, theoretically, well informed, but practically are so deficient that they have come to us from Bonn, Heidelberg, etc., to learn to put on a rubber dam.

*Dr. Jenkins*—I would like to ask Dr. Miller if he has found the students from the Real Schule superior to those from the Gymnasium. I have been informed that Dr. Esmarch formerly preferred pupils from the Gymnasium, but later has given a decided preference to those from the Real Schule. Also, what lectures do the dental and medical students have in common ?

*Dr. Miller*—The dental and medical students attend together lectures on anatomy, physiology and materia medica, but the former are not examined so strictly as the latter—in anatomy, for instance, on the head and neck only. He makes, on the average, a better examination than the medical student. I have not been able to discover any difference between those students who come from the Lyceum and those who come from the Real Schule, but I have found that students from Finland surpass all others in practical work.

*Dr. Patton*—Perhaps some one ought to explain that the course of study in the Lyceum is intended for professional men; that in the Real Schule for business.

*Dr. George*—One can but feel mortified when he compares the status of American schools with those of England. Surely our schools can be improved, or at least raised to the highest present standard—that of Harvard, for instance. We cannot, perhaps, do much as a body, but singly, by correspondence, we may do much to encourage men who have already devoted themselves to that object.

*Dr. Field*—Sir John Tomes sums it all up when he says, "It is one thing to know the scientific principles of an art, but it is quite another to carry them into effect. This requires an amount of skill of hand which can be attained only by long and careful practice under competent teachers." The curricula are thorough enough, and they all require enough, but the principal thing in dentistry is the practical part. With all the thoroughness of English courses of study, there is not so much accomplished as in American schools, because in the latter there is vastly more attention paid to the clinical work. I have lately read an article on Dental Education, pointing out that the English are retrograding in mechanical art, that the Americans are advancing in it, and the Germans are learning it through the Kindergarten. I would like to ask Dr. Miller if there is any possible basis for this statement.

*Dr. Miller*—Well, I don't know. Little children have building blocks, figures to draw, outlines to make, patterns to braid with fancy papers, and perhaps it has an effect on later progress in dentistry. I do not mean to depreciate American schools. I always advise German students to go to an American school after having made their degree in Berlin. Still, I would under no circumstances write D. D. S. after my name in Germany, on account of the miscellaneous work done in diplomas in America.

*Dr. Patton*—I am glad to hear Dr. Miller speak so strongly on this subject, and think this Society should take some means to stir up the dentists in America. There can be no doubt that there are innumerable instances in which persons in Germany, using the title of American dentist, have been gone from home but three months. Dr. Petermann, of Frankfort, has written his opinions very frankly in the German magazines, and has accumulated indubitable evidence of false diplomas from Philadelphia and Baltimore.

*Dr. George*—This Society passed at one time a resolution bearing upon this subject. Can any one tell us exactly what it was?

*Dr. Miller*—Graduates of the Pennsylvania College, to become members of this Society, must read an original article on some subject connected with dentistry, and pass a special examination in the English language. We have a record of the foreign diplomas improperly granted, and the Pennsylvania school heads the list, with the Philadelphia a good second. Baltimore stands next, and New York is fourth. No other college has issued more than three. The Pennsylvania and Philadelphia colleges have done more harm here by awarding diplomas to unworthy persons than sham institutions have by selling them.

*Dr. Field*—There have been just such cases in England, and lately, too.

*Dr. Elliot*—I had great hopes, when I became a lecturer on Operative Dentistry, of being able to accomplish good work in this direction. But after five years I resigned, disheartened. Attendance on the lectures was not obligatory, and I had, on the average, about fifteen. You all know that we have now the registration law in England, but, after all, it seems liable to fail. At any rate, it is not enforced in busy London, as the example of a flourishing American firm of bridge and crown workers will show. The firm advertises extensively, and they really are not eligible to practice, not being registered, not having diplomas from an English college, nor from Michigan or Harvard, or if from these two colleges, not having had ten years' practice after graduation, as is required by law. This is a point which may not be known to all of you.

*Dr. Field*—I had exactly the same experience in the same position that Dr. Elliot held, and I withdrew after three months. I had an attendance at my lectures of only six.

*Dr. Elliot*—I suppose every one knows of the Odontological Society of Great Britain. I proposed to them to get up some clinics. Their reply was that they were a scientific body, and wanted nothing to do with practice. I arranged two clinics at my own house, and invited the Society to see, at the same time, Dr. Rosenthal's apparatus for the treatment of alveolar abscess. There are one hundred members of the Society. Five came.

The President announced the Section of Mechanical Dentistry, and called on the chairman, Dr. Patton.

*Dr. Patton*—I have prepared no paper, as there have been no really great strides made in mechanical dentistry since one year ago.

Dr. Kingsley presented the Knapp blow-pipe, but the pressure of gas was insufficient to make it a perfect success.

Dr. Bryan presented a variety of bridge-work models.

*Dr. Jenkins*—I wish to present a new material for taking impressions, particularly where there are heavy undercuts. It is an impure variety of gutta-percha. My attention was called to it by Dr. F. H. Young, who has been experimenting with all sorts of gutta-perchas for two years, and finds this the best for the purpose. It must first be softened in hot water, put smoothly into the impression cup and placed in the mouth at a temperature such that the hand will barely perceive that it is warm. It is left in the mouth from four to five minutes, taken out quickly, held in the hand until the disarranged parts resume their places, which they will invariably do, and then suspended in cold water in such a way that no part of the still soft impression shall touch the vessel containing the water. In separating, it must not be made too hot, but allowed to be in the water ten or twelve minutes, when it may be separated easily, and if done properly another model can be made from the same impression.

*Dr. Förberg*—I think Dr. Suersen uses the same gutta-percha for his operations.

*Dr. Patton*—Zahnarzt Kellner, of Cologne, takes beautiful impressions by laying a mixture of wax and gutta-percha over a wax impression, and then repeating the process.

*Dr. George*—Dr. Herbst has sent a few models illustrating his method of making small partial plates without taking an impression. He presses a piece of rubber into the mouth as you see illustrated by these models, takes it out, trims it, sets up the teeth on it, tries it in and vulcanizes.

A few months ago, having considerable annoyance from the rubber we had been using for plates, I instituted a series of experiments for the purpose of determining what was the best varieties for our purposes. I regret that at present I can give no full report. Many little difficulties have turned up unexpectedly, each requiring considerable time and patience to overcome, so that my progress has been nothing like so rapid as I anticipated. I have been testing for elasticity and fracture strength. I have found thirty-seven samples of rubber in the market. I have taken twenty pieces of each variety, of the dimensions of 5 by 9 by 1 millimeters, and tested each of these pieces with a very simple little machine made for the purpose. I hope next year to make a complete report.

*Dr. Miller*—I should think that a tough rubber would be better for plates, and an elastic one for clasps.

*Dr. Field*—Were these specimens made under pressure ?

*Dr. George*—No. They were cut, placed between plates of glass, plaster of Paris was flowed around them and they were then vulcanized.

(TO BE CONTINUED.)

